# A Single Step *in vitro* Bioassay Mimicking TLR4-LPS Pathway and the Role of MD2 and CD14 Coreceptors

**DOI:** 10.3389/fimmu.2020.00005

**Published:** 2020-01-24

**Authors:** Pramod Jagtap, Puja Prasad, Abhishek Pateria, Sachin D. Deshmukh, Shalini Gupta

**Affiliations:** ^1^Department of Chemical Engineering, Indian Institute of Technology Delhi, New Delhi, India; ^2^Center for Sepsis Control and Care, Jena University Hospital, Jena, Germany

**Keywords:** TLR4, MD2, CD14, LPS, ligand screening, *in vitro* bioassay

## Abstract

Acute systemic Gram-negative bacterial infections are accompanied by release of lipopolysaccharide (LPS) endotoxins into the bloodstream and an innate immune host response via the well-known toll like receptor 4 (TLR4) pathway. In this, LPS associates non-covalently with TLR4 to form an activated heterodimer (LPS/MD2/TLR4)_2_ complex *in vivo*, assisted by a coreceptor CD14. This complexation process has been illustrated *ex vivo* using indirect methods such as cytokine, interleukin, TNF-α measurements and by direct demonstration of sequential binding events on a surface using advanced optics. We are the first ones to carry out homogeneous self-assembly of LPS-rTLR4-MD2 conjugates *in vitro* in a single step, and further demonstrate the role of CD14 as a catalyst during this process. The assay comprises of LPS, MD2, CD14, and recombinant TLR4-conjugated magnetic particles co-incubated in a buffer at room temperature. The complexes are removed by magnetic separation and the extent of binding is estimated by quantifying the unbound biomolecules in the supernatant using standard biophysical techniques. Our results show that rTLR4-MD2-LPS complexes form in an hour and follow a 1:1:1 stoichiometry, in agreement with the *in vivo/ex vivo* studies. The assay is also highly specific; addition of known LPS-binding ligands decreased the LPS-rTLR4 complexation, allowing its use as a rapid tool for molecular inhibitor screening.

## Introduction

Toll like receptors (TLRs) are a family of pattern recognition receptors (PRRs) whose name was first coined by scientists Volhard and Wieschaus in the year 1985 due to the structural similarity of these class of molecules with a protein encoded by the toll gene present in drosophila (1). These protein molecules are present on the surface of immune cells, more specifically on macrophages and dendritic cells, and are responsible for the activation of innate immune response inside a host (2). Among the TLRs, TLR4 is a type I integral membrane glycoprotein that is specifically produced against Gram-negative bacterial endotoxins lipopolysaccharides (LPS). These LPS molecules exist in million copies per cell and are released into the bloodstream, in the form of random clusters or outer membrane vesicles (OMVs), as the cells multiply or die in response to antibiotic treatment (3, 4). It is now well-established that activation of TLR4 is one of the two innate immune pathways triggered during Gram-negative bacterial sepsis in immunocompromised patients (the other one is caspase 11 activation in macrophages) (5, 6). Sepsis is defined as a life-threatening condition that leads to circulatory abnormalities, organ dysfunction, and death due to the overwhelming response of the body's immune system to bloodstream infection (7, 8). Recently, a few studies have demonstrated that the repetitive exposure of pathogen associated molecular patterns (PAMPs) such as LPS to innate immune cells can lead to a subsequent increase or decrease in their responsiveness termed as training or tolerance, respectively (9). These mechanisms are now being studied in septic conditions and seem to indicate highly complex TLR4 signaling in the human body (10).

TLR4-LPS complexation is an important event in the activation of innate immune cascade and the production of NF-κB and mitogen-activated protein kinases (11). During this process, myeloid differentiation-2 (MD2) and membrane cluster differentiation-14 (CD14) coreceptors play an important role by taking part in the binding process and promoting complex formation (12). The CD14 protein is available in two forms, a soluble form and a glycosylphosphatidylinositol-anchored membrane form (13). While the role of membrane CD14 in TLR4-LPS complexation process has been studied extensively, the role of soluble CD14 still remains unclear (14). Similarly, MD2 is a small cysteine rich glycoprotein that binds with the ectodomain of the TLR4 molecule (15). TLR4-MD2 heterodimer formation is necessary for LPS binding and further toll interleukin receptor (TIR)-domain-containing adapter-inducing interferon-β (TRIF)-dependant signaling pathway (11).

A number of *in vitro* and *ex vivo* gene manipulation studies have proven the role of TLR4 and MD2 in LPS responsiveness (16–18). Segal et al. reported that once MD2-LPS forms a stable complex, it causes TLR4 activation in a CD14 and LPS-independent manner (19). Kim et al. isolated the hybrid crystal structure of TLR4-MD2-LPS in Hi5 insect cells which illustrated that MD2 binds to the concave N-terminal segments and central domains of the TLR4 molecule. They further proposed the model of LPS-induced dimerization (20). Recently, the entire TLR4-LPS cascade was reconstructed by Riu et al. using total internal reflection fluorescence (TIRF) microscopy in which, LPS transfer from lipoprotein binding protein (LBP) to membrane CD14, and membrane CD14 to TLR4-MD2 complex was beautifully demonstrated (21). A number of other researchers have also successfully demonstrated TLR4-LPS complexation and stoichiometry using *in vivo* experiments, X-ray crystallography, and *in silico* methods (20, 22–24). There are still, however, some ambiguities in the field regarding the role of serum/soluble CD14 in TLR4-LPS complex formation and the sequence of rTLR4, MD2, LPS binding. Also, no simple *in vitro* model exists that can directly provide information about the mode, stoichiometry and kinetics of molecular interactions and to further screen ligands for developing new therapeutics targets for sepsis management.

**Figure 1:**
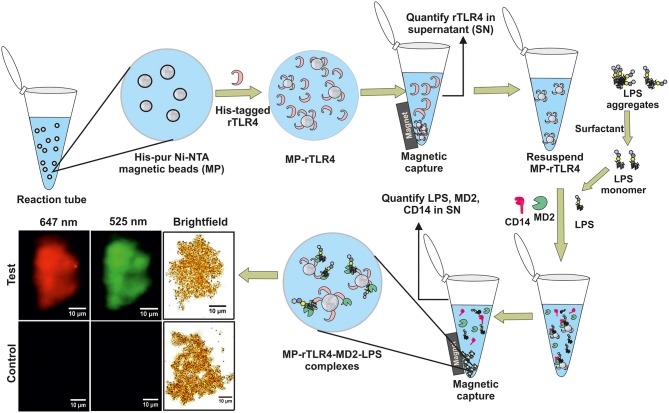
A schematic representation of our experimental approach showing rTLR4 conjugation to magnetic nanoparticles and its *in vitro* complexation with LPS in the presence of free forms of MD2 and CD14 co-receptors. The bottom left panel shows the actual optical micrographs of MP-rTLR4-MD2-LPS (test) and MP-rTLR4 (control) complexes recorded in brightfield and complementary fluorescence modes at two different emission wavelengths: 525 nm (green) for alexafluor-tagged LPS and 647 nm (red) for alexafluor-tagged MD2.

We have designed a simple and robust *in vitro* bioassay that mimics the TLR4-LPS recognition pathway in a single step and provides outcomes in an hour. The assay works by mixing all the essential building blocks required for biological complexation, i.e., LPS, MD2, CD14, and magnetically-tagged TLR4, together in a single reaction tube and isolating the formed complexes using a simple magnet (Figure 1). The unbound components that remain in the supernatant are analyzed using standard biophysical techniques to provide information about the extent of reaction and hence, its stoichiometry and kinetics. A detailed optimization study has been carried out to determine the best operating conditions for obtaining an enhanced assay performance. In addition, the assay has been applied for screening of TLR4 pathway inhibitory ligands to demonstrate its clinical utility. Our overall findings provide a mechanistic understanding of the relative importance and role of the different coreceptors involved in the TLR4-LPS complexation process.

## Materials and Methods

### Materials

Human recombinant toll like receptor 4 (rTLR4) (R & D Systems, USA); Recombinant human myeloid differentiation-2 (MD2) protein (Abcam, USA); His-tagged human cluster differentiation-14 (CD14) protein (Sino Biologicals, USA); Sushi peptide of purity > 95% [HAEHKVKIGVEQKYGQFPQGTEVTYTCSGNYFLMC (M.W. 4083 Da)] (Genemed Synthesis, USA); Purified LPS-alexafluor@488 conjugate (M.W. 10 kDa) extracted from 055:B5 *Escherichia coli* (*E. coli*) (Molecular probes, Life Technology, USA); Vancomycin hydrochloride (M.W. 1485.7 Da) (Himedia Pvt. Ltd, India); Tween 20, Pierce^™^ LAL chromogenic endotoxin quantitation kit, alexafluor 647 microscale protein labeling kit, hisPur^™^ Ni-NTA 1 μm magnetic beads (MPs) and magnetic separation rack (Thermofisher Scientific, India); Alexidine dihydrochloride (M.W. 581.7 Da), bacitracin (M.W. 1422.7 Da), tris HCl, phosphate buffer saline (PBS), 4-(2-hydroxyethyl)-1-piperazineethanesulfonic acid (HEPES), imidazole and sodium phosphate (Sigma Aldrich, India); Pam3CSK4 rhodamine and Poly (I:C) fluorescein (InvivoGen, USA); Sodium chloride (NaCl), sodium hydroxide (NaOH) and HCl (SRL, India); Milli-Q water (resistivity ~ 18 MΩ.cm) (Millipore, India). All reagents were of analytical grade and used at room temperature (RT). The 10 mM PBS buffer was prepared at pH 7.4.

### Methods

All the experiments were performed in triplicate and the results were plotted as their mean ± 1 standard deviation (SD). One way ANOVA analysis were carried out to find the statistically significant differences between two data sets (*P* < 0.05; Tukey's test). All concentrations were reported in molar units for ease of calculations knowing well that LPS is heterogeneous and it is difficult to establish its molecular weight without separating its fractions. The average molecular weights used in these calculations was 10 kDa as recommended by the manufacturer.

#### Preparation and Characterization of rTLR4-Functionalized MPs

Ten microliters of 1.25 mg/mL MPs were washed twice with 200 μL of equilibration buffer. The equilibration buffer comprised of 100 mM PBS, 0.6 M NaCl, 0.05% Tween 20 and 30 mM imidazole and its pH was adjusted to 8.0 using 1 M NaOH. The MPs were separated using a magnetic removal rack. The washed beads were mixed with 200 ng of rTLR4 in a total reaction volume of 200 μL equilibration buffer and gently mixed over a rotospin test tube mixer (Tarsons) at 50 rpm for 1 h at RT. The MP-rTLR4 composites were then removed using the magnetic separation rack and washed twice with 200 μL of equilibration buffer. The particles were finally resuspended in PBS and stored at 2–8°C until further use. The entire procedure was repeated with 400 and 600 ng of rTLR4 keeping all other conditions constant and the supernatants collected from each run were analyzed for quantifying the unconjugated rTLR4.

For particle size and zeta potential characterization, the MP-rTLR4 suspensions were diluted 10x with MilliQ water and analyzed using Zetasizer nano ZS90 (Malvern Instruments Ltd.) Simultaneously, the collected supernatants were also concentrated using the 30 kDa MWCO filters (Millipore) using centrifugation at 3500 RCF and ~ 20 μL of these samples were analyzed using the standard western blot (25) and silver staining techniques (26) for protein quantification. The band intensities obtained in both methods were quantified using ImageJ software (version 1.46r) (see Figure S1 and Supplementary Information for further details).

#### Performance of LPS-rTLR4 Reaction

*(a) LPS monomerization:* A 100 μM master stock was prepared by dissolving 100 μg of lyophilized LPS powder in 500 μL of PBS. 100 μL of this stock was further diluted to 1 mL to obtain a 100 nM working solution. Varying amounts of Tween 20 were added to 200 μL of this LPS solution (concentrations ranging from 0.1 to 1.5 v/v %) and their fluorescence intensities were measured at λ_ex_ = 480 nm and λ_em_ = 525 nm using Spectramax i3X multimode plate reader (Molecular devices).

*(b) MD2 and CD14 labeling with alexafluor 647*: A 25 μL aliquot of 1 mg/mL protein was taken in a reaction tube and mixed thoroughly with 2.5 μL of 1 M sodium bicarbonate. To this, 2 μL of a freshly prepared 7.94 nmol/μL reactive dye solution was added and incubated at RT for 15 min. The reaction mixture was purified using a resin column to remove the unreacted dye. The extent of protein labeling was estimated by measuring the absorbance at A_280_ and A_650_ nm and applying a correction factor of 0.03 to correct for the fluorophore's contribution at 280 nm. The average number of alexafluor 647 tags per CD14 and MD2 molecules was estimated to be 1 and 3, respectively. The purified protein was finally resuspended in PBS and stored at 2–8°C until further use.

*(c) LPS-rTLR4 reaction:* 0.5 μM stock solutions of MD2 and CD14 each were prepared by reconstituting specified amounts of lyophilized powder in PBS. For preparing multiple combinatorial sets of reactions, different concentrations rTLR4-conjugated MP, MD2, CD14, and LPS were mixed in a 200 μL volume of PBS containing 0.5% Tween 20 (referred to as 0.5% PBST from hereon). The reaction components were co-incubated at RT under gentle vortexing for 1 h followed by their magnetic separation using the magnetic rack. MP-rTLR4 complexes were then washed twice with 200 μL of equilibration buffer and resuspended in PBS. The amounts of unreacted LPS and MD2 were quantified by mixing 90 μL of the supernatant with 110 μL of 0.5% PBST in a black nunc plate followed by mixing in an orbital shaker for 15 min at 150 rpm and 37 °C. The fluorescence intensity of this mixture was recorded using Spectramax i3X plate reader simultaneously at two different wavelengths (λ_ex_ = 480 nm, λ_em_ = 525 nm for LPS and λ_ex_ = 633 nm, λ_em_ = 647 nm alexafluor 647 tagged MD2) and their amounts in the supernatant were determined from their respective calibration graphs (see Supplementary Information).

The CD14 in the supernatant was quantified using two routes. First, by pre-concentrating the samples to 20 μL followed by their silver staining using the same procedure described above for rTLR4 quantification. Second, by tagging the CD14 (instead of MD2) with alexafluor 647 and measuring its fluorescence intensity via the iMax multimode plate reader. The MP-rTLR4-MD2-LPS complexes were also visualized directly in brightfield and fluorescence modes at two different emission wavelengths (525, green and 647 nm, red) using the Olympus BX-53 optical microscope. For this, 10 μL of the concentrated complex suspensions were mounted on a glass slide and focused using the 100X oil immersion objective. The images were taken with an Olympus E3 CCD color camera in the manual exposure mode (target: 120, time: 350 ms, gain: 1.2). For kinetic measurements, supernatants were collected at 15, 30, 45, 60, and 90 min from independent reaction sets and analyzed for CD14, MD2, and LPS concentrations.

*(d) TLR4 pathway inhibitory ligand screening:* Four different ligand were screened using this assay model. Each ligand added in a separate reaction mix at a concentration of 2.4 μM which was close to their minimum inhibitory concentration (MIC)–alexidine (MIC 1.2 μM) (27), bacitracin (MIC 3 μM) (28), vancomycin (MIC 1 μM) (29), and sushi peptide (MIC 1.5 μM) (30). The effect of ligand addition was investigated by plotting the LPS fluorescence intensity in the supernatant for 50 nM LPS solutions in the presence of ligand before and after adding the optimum amounts of MP-rTLR4, MD2, and CD14 (rTLR4:MD2:CD14::10:20:10) also collectively known as the assay mix.

#### LPS Quantification Using LAL (Limulus Amoebocyte Lysate) Assay

Endotoxin concentrations in the supernatant were also measured using the chromogenic LAL assay. For this, a microwell plate was pre-equilibrated in a heating block for 10 min at 37 ± 1°C. To each well, a 50 μL volume of either the standard or the unknown sample was added followed by immediate addition and mixing of 50 μL of the LAL reagent. The plate was then incubated on the heating block for an additional 10 min at 37 ± 1°C followed by injection of 100 μL of the chromogenic substrate (pre-warmed to 37 ± 1°C) to each well. The incubation process was continued for another 6 min and then, a 100 μL volume of the stop reagent (25% acetic acid) was added and mixed thoroughly. Finally, the plate absorbance was measured at 405–410 nm using the iMax multimode plate reader. A standard linear curve was generated in the 10–100 pg/mL concentration range using the *E. coli* endotoxin standards included in the kit, and the endotoxin levels in the unknown samples were interpolated from this curve.

## Results and Discussion

### Assay Preparation

**Figure 2:**
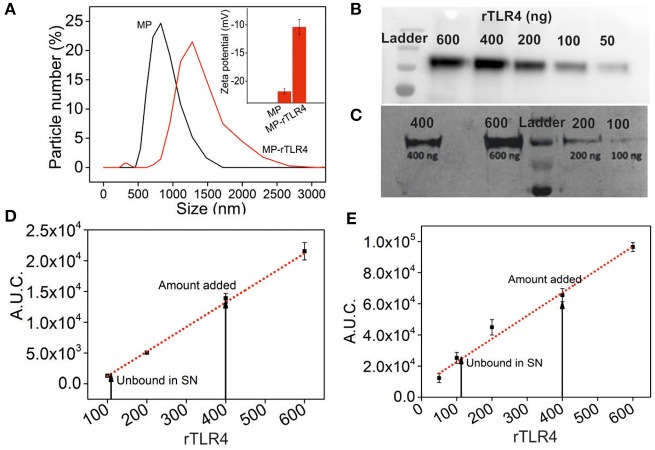
Characterization of rTLR4 binding to MPs. **(A)** A dynamic light scattering plot showing the hydrodynamic particle size distribution and the expected size increase of magnetic nanoparticles before and after rTLR4 conjugation (rTLR4:MP::10:431 g/g). The inset shows the corresponding change in surface charge potential. Typical gel patterns obtained for the supernatant using **(B)** western blot and **(C)** silver staining after magnetic removal and their respective rTLR4 calibration charts, **(D,E)** obtained after quantifying the color intensity of each block and integrating the area under curve (A.U.C.) (*n* = 3).

Our assay was based on the simple principle of multimolecular complex formation and removal, followed by analysis of the supernatant composition. In order to facilitate this process, we conjugated rTLR4 to MPs to remove the complexes using a simple magnet. Positively charged Ni-NTA MPs were used for this purpose in order to bind them with the his-tagged rTLR4 proteins using a well-known chelation chemistry (31). The particles were mixed with TLR4 in three varying ratios (125:2, 125:4, 125:6 w/w) and the excess protein was removed by washing the particles at the end of the incubation cycle. The amount of rTLR4 conjugated to MPs was then estimated by quantifying the supernatant (obtained after the magnetic separation step) using western blot and silver staining techniques. The typical bands obtained during the rTLR4 calibration process and their respective quantitation plots obtained by integrating the block intensities are depicted in Figures 2B–E. Using these plots, the unknown rTLR4 concentrations in the supernatant, and in turn their respective rTLR4-MP binding efficiencies, were determined for all the three cases. The maximum binding efficiency of 78 ± 5% was achieved with 125:4 w/w whereas, 125:2 and 125:6 w/w showed lower values of 39 ± 5 and 58 ± 5%, respectively. Thus, 125:4 w/w ratio was chosen for all the subsequent rTLR4 conjugation reactions. At this optimum condition, the average molar ratio of rTLR4 to MP was estimated to be 300,000:1. As a result, not only did the hydrodynamic diameter of the conjugated MPs increase from 1 ± 0.05 to 1.4 ± 0.05 μm (Figure 2A), the zeta potential also dropped from being more to less negative as the negatively charged nitrilotriacetic acids (NTA) present on the MP surface got masked by the less negatively charged rTLR4 proteins.

**Figure 3:**
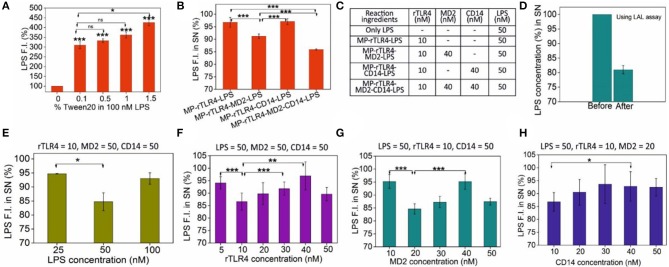
**(A)** LPS monomerization aided by surfactant Tween 20 addition. **(B)** The importance of CD14 and MD2 on the extent of complexation, and **(C)** the molar amounts of molecules used in these reactions. **(D)** Cross-validation of LPS capture using the LAL assay. **(E–H)** Sequential scouting experiments: Bulk concentrations of LPS **(E)**, rTLR4 **(F)**, MD2 **(G)**, and CD14 **(H)** were optimized one-by-one and the optimized values were fixed in all the subsequent experiments to get maximum LPS capture efficiency at each step. The fixed concentration values of the remaining reagents (all in nM) are listed on top of each graph. All experiments were performed in 0.5% PBST, pH 7.4 (*n* = 3) (SN = supernatant, ns, *p* >0.05, *p = 0.01–0.05, **p = 0.001–0.01, ***p < 0.001).

Once the rTLR4-MP conjugates were successfully prepared, next we optimized the assay parameters for our aimed rTLR4-LPS complexation reaction. For this, we first investigated the stability of the LPS fluorescence intensity in three different buffers. This was necessary as the LPS fluorescence intensity was the prime indicator of the extent of rTLR4-LPS-MD2 complexation reaction. While PBS showed exceptional fluorescence stability over 1 h, the signal quality gradually decreased in HEPES and tris HCl with time (Figure S2). Thus, PBS was finalized as a suitable reaction medium for all subsequent rTLR4-LPS reactions. Another important aspect was LPS monomerization. LPS being an amphiphilic molecule prefers to stay in an aggregated state in aqueous solutions which leads to the quenching of its fluorescence intensity above its critical micellar concentration (CMC approx. 1.3 to 1.6 μm in case of *E. coli*) (32). *In vivo*, this is taken care of by the lipid binding protein (LBP) which monomerizes the LPS molecule by forming a high-affinity complex with LPS (K_D_ ~ nM) before transferring it to CD14 (33, 34). We decided to use a simple non-ionic surfactant Tween 20 to monomerize LPS based on our previous experience (35). This surfactant can closely associate with the long alkyl chains of LPS molecules via hydrophobic interactions and disrupt the LPS aggregates (36). When different amounts of Tween 20 were added to the PBS medium, the LPS fluorescence intensity was seen to increase commensurately with the surfactant concentration in the range studied (0.1–1.5 w/v%) (Figure 3A). Based on these results, we fixed our surfactant concentration at 0.5 w/v%, as higher amounts carried the risk of protein destabilization and solution foaming at the cost of very little gain in fluorescence signal. It is important to note that in the absence of any Tween 20, the rTLR4-LPS complexation reaction showed an irregular trend (see Figure S3) suggesting the essential role of surfactant in the entire process.

### Assay Performance and Optimization

With the particles prepared successfully and the media fully optimized, we finally carried out the rTLR4 complexation reactions with LPS in the presence of CD14 and MD2 coreceptors. All the reagents were co-incubated into a single reaction tube at RT for 1 h and the supernatant was analyzed to determine the extent of LPS capture. For the various reaction combinations studied, the only parameter that was monitored was the LPS fluorescence intensity in the supernatant as the main objective here was to determine the role of MD2 and CD14 coreceptors in the complexation process. The experiments were performed in three modes–(1) without any coreceptor, (2) with only one of the two coreceptors and (3) with both coreceptors as shown in Figures 3B,C. The results obtained suggested that while both coreceptors were beneficial for the overall complexation process resulting in maximum LPS capture, it was MD2 that was absolute necessary for the reaction to take place, and CD14 played only a complementary role and did not directly take part in the reaction. These findings are in accordance with the *in vivo* and mammalian cell studies reported in the literature (23, 37). The assay was also highly specific; replacing LPS with TLR1/2-binding ligand Pam3CSK4 or TLR3-binding ligand Poly (I:C) had little impact on the supernatant fluorescence intensity (Figure S4).

Since the concentrations of the reagents were somewhat randomly chosen in the above experiments to only understand the overall trends and behavior of the molecules involved, we next proceeded to a full optimization study to determine the most optimum bulk molecular concentrations required for maximum rTLR4-LPS complexation. To this end, detailed scouting experiments were performed in which each biomolecule type was sequentially optimized while keeping the concentrations of the remaining ones constant. For instance, as shown in Figure 3E, rTLR4, MD2, and CD14 concentrations were fixed at 10, 50, and 50 nM, respectively while the LPS concentration was varied from 25 to 100 nM. The best results were obtained for 50 nM LPS and so, this LPS concentration was fixed for all subsequent experiments. Here, the maximum LPS retention rate of ~15% may appear low in the first glance but it is actually not less considering that the relative molar ratios of TLR4 and LPS used in the experiments were 10 and 50 nM, respectively (or, a maximum achievable retention rate of 20% assuming 1:1 stoichiometry). However, the reason why the LPS capture reduced upon its further increase is not fully clear to us and is attributed to entropic effects that are often observed in similar nanoparticle-based capture studies (32). Experiments were repeated in a similar fashion for rTLR4 (Figure 3F), MD2 (Figure 3G) and CD14 (Figure 3H) and in the end, the optimum molar ratio for maximum LPS capture was found to be LPS:rTLR4:MD2:CD14::5:1:2:1. It is important to note that this is the *bulk* molar ratio, not the molar stoichiometry of the molecules in the bound form.

### Assay Kinetics

**Figure 4:**
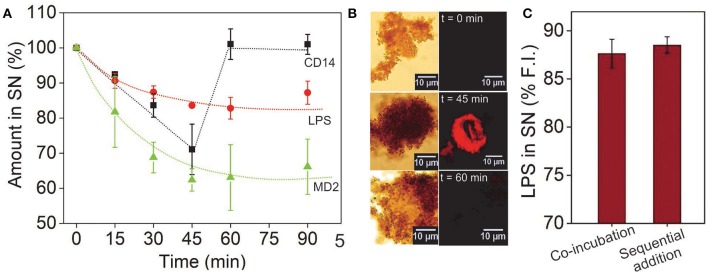
**(A)** Time-lapsed measurements of LPS, MD2, and CD14 concentrations showing molecular binding kinetics and the role of CD14 as a catalyst. While the LPS and MD2 molecules were measured using their fluorescence intensity in the SN, CD14 was quantified via silver staining. **(B)** CD14 visualized after alexafluor tagging (λ_em_ = 647 nm). The complementary brightfield and fluorescence optical images showed CD14 in the bound state only in the middle of the reaction cycle. **(C)** Varying the sequence of molecular addition had little impact on the extent of LPS capture. Here, sequential addition implies prior incubation of CD14 with LPS and MP-rTLR4 with MD2 followed by their rapid mixing as opposed to co-incubation of all the reagents from the beginning (*n* = 3) (SN = supernatant).

To determine how long it takes for the reaction to complete, we performed a kinetic study in which the rTLR4-LPS binding was monitored over 90 min by measuring the amounts of LPS, MD2 and CD14 remaining in the supernatant (or conversely, consumed in the reaction) as it progressed forward (see Figures S5A, S6, S7). The LPS and MD2 concentrations were quantified via fluorescence spectroscopy measurements at two different wavelengths and CD14 was quantified using the silver staining technique. The results plotted in Figure 4A illustrated that the concentrations of all the three reagents reduced monotonically in the first 45 min, and while the LPS and MD2 amounts reached saturation in this time, CD14 concentration was fully regained in the supernatant and continued to remain so for the remaining period. This observation was interesting as it clearly demonstrated that both LPS and MD2 were confined in the bound state and were hence, essential components of the bioconjugation process, whereas, CD14 played a catalytic role during the rTLR4-LPS-MD2 complexation process. These results are in accordance with the recent findings of Ryu et al. (21). The stoichiometry of the bound rTLR4:LPS:MD2 complexes using our approach was found to be 1:1:1 which was again in agreement with the reported values in the literature (20, 38).

**Figure 5:**
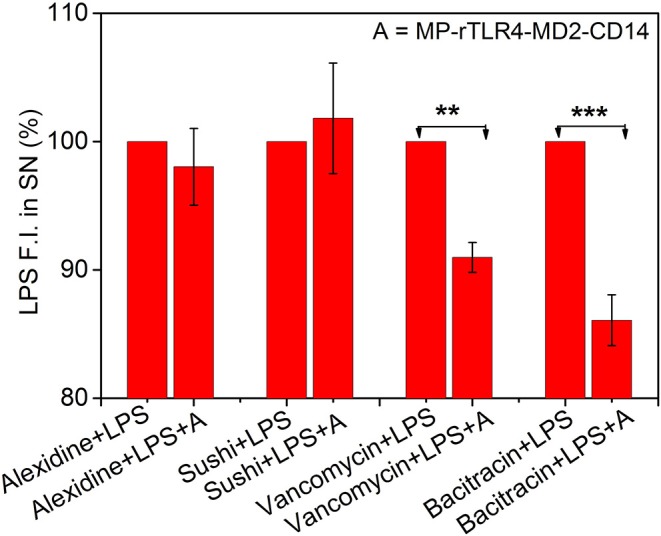
Inhibitory ligand screening. Results shown for LPS-interacting alexidine and sushi molecules used as test, and LPS-non-interacting bacitracin and vancomycin molecules used as control (*n* = 3). While the LPS fluorescence intensity in the SN remained constant upon addition of MP-rTLR4-MD2-CD14 assay mix (marked here as “A”) in the presence of alexdine and sushi, it reduced significantly with bacitracin and vancomycin suggesting the inhibitory potential of the first two molecules for obstructing rTLR4 pathway. All the data have been normalized with respect to the fluorescence intensity before “A” addition. The concentrations of each ligand used in reaction was 2.4 μM in their respective reactions (SN = supernatant, **p = 0.001–0.01, ***p < 0.001).

The accuracy of our results was further verified by tagging the CD14 molecules (instead of MD2) with a fluorescent dye to allow for their easy visualization via an optical microscope. The micrographs obtained showed a distinct appearance of red fluorescence (λ_em_ = 647 nm) up to 45 min that were no longer visible at 60 min (Figure 4B), reconfirming the transitory role of CD14 in the binding process. The fluorescence spectroscopy results were also validated using the well-known LAL assay which is an industrial gold standard for endotoxin quantification (39). The results for LPS using LAL assay again showed similar value (Figure 3D and Figure 5B) which matched our fluorescence data proving that our assay was indeed robust and reliable. Finally, to rule out any possibility of experimental design artifacts, we performed additional control experiments in which CD14 and LPS were incubated separately from MD2 and CD14 mixture and then mixed altogether. This was done with the understanding that, *in vivo*, LPS binds to the CD14 coreceptor first before being transferred to the MD2-TLR4 heterodimer. We wanted to ensure that the CD14-binding epitopes on the LPS molecules were not being sterically masked by MD2/rTLR4/(MD2-rTLR4) in our case due to the intrinsic differences in their binding rate constants. However, no significant difference in data was found between the two incubation procedures (Figure 4C) suggesting that our results for the rTLR4-LPS complexation process were as close to the real scenario as possible. On similar lines, Tan et al. extensively studied the oligometic structures, mydosomes as TLR4 recognition elements named as supramolecular organizing centers (SMOCs) and their role as subcellular site for TLR4 signals that played duel role in NF-kB activation and glycolysis. Also the programmable features of SMOCs were proposed for developing nanomedicine platform for immunomodulation (40). In this direction, our study can provide fundamental guidance for TLR4 complexation process and this information can be utilized for designing small molecules against TLR4 and its co-receptors.

### Assay Application for Inhibitory Ligand Screening

Finally, to demonstrate the clinical utility of our approach, we applied our optimized assay for the screening of TLR4 pathway inhibiting ligands. Four ligands were chosen for this purpose. Alexidine and sushi peptide were selected as positive controls due to their specific affinity toward LPS as shown in our recent studies (3, 4). Vancomycin and bacitracin were used as negative controls for their lack of reported interaction with LPS (41, 42). The inhibitory potential of the ligands was determined by evaluating the reduction in the LPS fluorescence intensity in the supernatant for LPS solutions in the presence of the affinity ligand and assay mix, with respect to those with affinity ligand alone (marked as 100%, see Figure 5). A smaller reduction in signal implied a more efficient inhibitor as it could compete with the rTLR4 molecule for LPS binding and hence, potentially disrupt the TLR4 pathway. Our results indicated that alexidine and sushi peptide were equally competitive in inhibiting rTLR4-LPS binding whereas, bacitracin performed least optimally. Vancomycin showed intermediate inhibitory behavior as the glycoside moieties present on this drug can interact with the lipid A portion of LPS (K_D_ ~ 0.5 μM) and inhibit its reaction (43, 44). Overall, our assay seemed to hold tremendous potential as a simple and quick screening tool for shortlisting new therapeutic candidates for sepsis treatment by leveraging the tightly-controlled molecular interactions in the TLR4 pathway.

## Conclusions

We have successfully demonstrated the fundamental aspects of the rTLR4-LPS complexation process including reaction time, stoichiometry and role of coreceptors using a simple and single step fluorescence assay designed to mimic the complex sequential *in vivo* pathway. The rTLR4-MD2-LPS stoichiometry was found to be 1:1:1 and the insoluble form of CD14 was seen to play a catalytic role during the complexation process. The entire assay was complete within 45 min and showed good screening potential for testing LPS-binding TLR4 pathway inhibitory ligands. While alexidine and sushi peptide showed perfect inhibition, vancomycin and bacitracin showed only partial inhibitory activity against LPS in that order. The assay, however, did not show the expected response with polymyxin B sulfate (data not shown) in spite of having known LPS sequestration activity. This leads us to conclude that the assay may be used as an initial quick tool for ligand screening and interaction followed by more advanced biophysical techniques to confirm the activity of the selected candidates, however, it may not be applicable to all molecules as the results may vary depending on the mechanism of ligand interaction with LPS and/or any of the other components present in the medium. Synthesis of more potent and specific molecular drug ligands for potential sepsis treatment and their cytotoxicity studies are currently underway in our group. The sensitivity of the assay may also be improved by using smaller magnetic nanoparticles to increase the effective number of LPS binding sites or by changing the TLR4 conjugation chemistry to include linkers for reducing steric hindrance.

## Data Availability Statement

All datasets generated for this study are included in the article/Supplementary Material.

## Author Contributions

PJ and PP designed the experiments. PJ performed the experiments, wrote the manuscript, and drew the figures. PP and AP assisted PJ in the experiments. SG and SD conceptualized the idea, provided overall supervision and feedback for the research work, writing, and data interpretation. All authors have reviewed the manuscript for final approval.

### Conflict of Interest

The authors declare that the research was conducted in the absence of any commercial or financial relationships that could be construed as a potential conflict of interest.
