# Supplementary material for: A Single Step in vitro Bioassay Mimicking TLR4-LPS Pathway and the Role of MD2 and CD14 Coreceptors
Source: Front Immunol. 2020 Jan 24;11:5. doi: 10.3389/fimmu.2020.00005 (PMC6992608; doi:10.3389/fimmu.2020.00005)
Supplement: Supplementary file 1 [file Data_Sheet_1.PDF]

A single step *in vitro* bioassay mimicking TLR4-LPS pathway and the role of MD2 and CD14 coreceptors

Pramod Jagtap[1], Puja Prasad[1], Abhishek Pateria[1], Sachin Deshmukh[2] and Shalini Gupta[1,*]

[1]*Dept. of Chemical Engineering, Indian Institute of Technology Delhi, New Delhi, India 110016*

[2]*Center for Sepsis Control and Care, Jena University Hospital, Jena, Germany 07747*

Supplementary Information

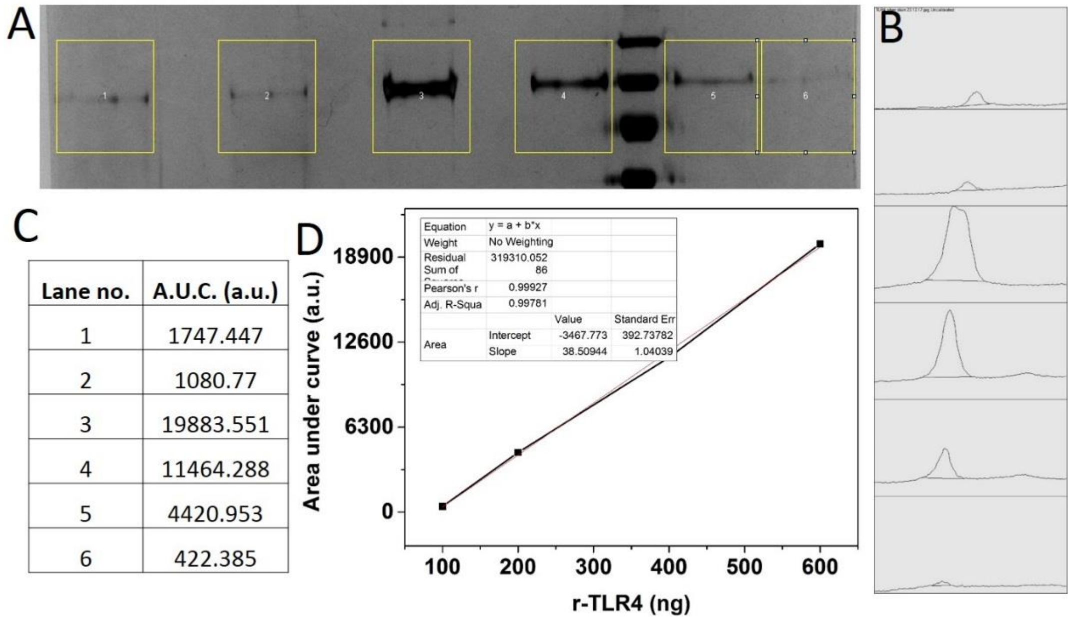

Fig. S1. **(A)** A typical gel image obtained after silver staining procedure and the respective area selected for analysis using a rectangular box. **(B)** Plots and **(C)** area under curve of obtained for the respective bands. **(D)** The final calibration curve for rTLR4 concentration.

Steps followed for protein quantitation from gels using ImageJ:

1. After silver staining the gel/western blot, the image of the gel was captured using Gel doc and saved in the JPEG/TIFF format. The brightness and contrast were adjusted if the image was too light.
2. The file was opened in ImageJ (version 1.46r) and the rectangle tool was selected and drawn around the lane, making sure that some of the empty gel between the lanes and the white space outside of the band was also selected.
3. In the "analyse gel" tab, the first lane was selected.

4. After selecting the first lane, the rectangle was moved to the next lane.
5. This second step was repeated until all lanes were marked and numbered.
6. The area under the curve was then calculated using the magic tool.

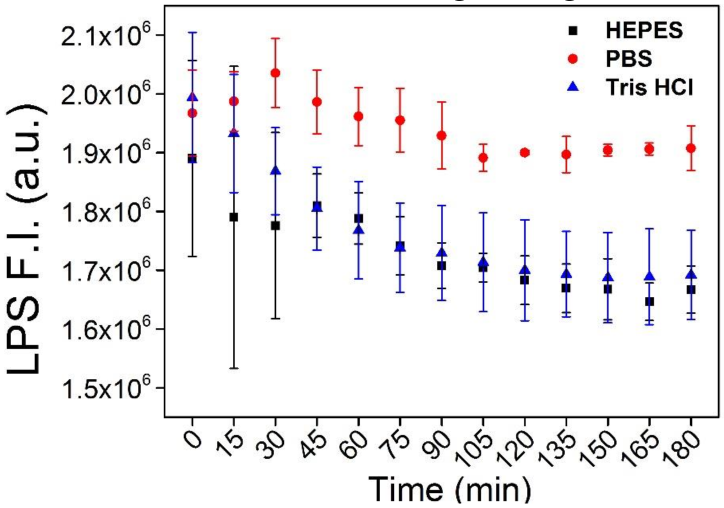

Fig. S2. Buffer selection for rTLR4-LPS reaction. The LPS fluorescence intensity of LPS was monitored in 40 mM HEPES pH 7.4, 10 mM tris HCl pH 7.4 and PBS over 3 h (n = 3). PBS showed the most stable and time-invariant fluorescence intensity values and was selected as our buffer of choice for all experiments.

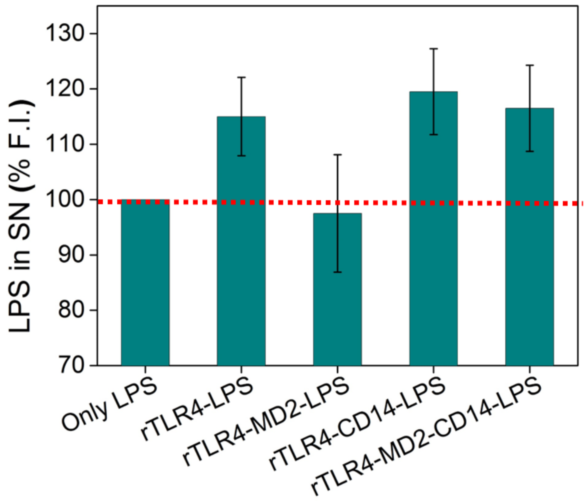

**Reaction ingredients**	**MNP-TLR4 (nM)**	**MD2 (nM)**	**CD14 (nM)**	**LPS (nM)**
Only LPS	-	-	-	50
MNP-TLR4-LPS	10	-	-	50
MNP-TLR4-MD2-LPS	10	40	-	50
MNP-TLR4-CD14-LPS	10	-	40	50
MNP-TLR4-MD2-CD14-LPS	10	40	40	50

Fig. S3. Extent of LPS binding in PBS without Tween 20 and in the presence of different coreceptors. The result showed unusual behaviour as the LPS fluorescence intensity increased erroneously (> 100 %) instead of decreasing upon rTLR4-LPS complexation. The respective concentrations used in these reactions are mentioned in the

table. (n = 2).

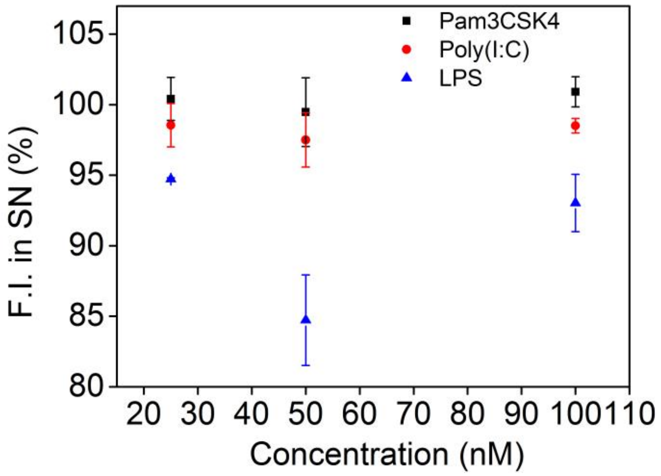

Fig. S4. Negative control experiments performed with fluorescently-tagged Pam3CSK4 (TLR1/2-binding ligand) and Poly (I:C) (TLR3-binding ligand) to determine assay specificity. Constant fluorescence intensities in the SN indicated no interaction of rTLR4 with either Pam3CSK4 or Poly (I: C) (n = 2).

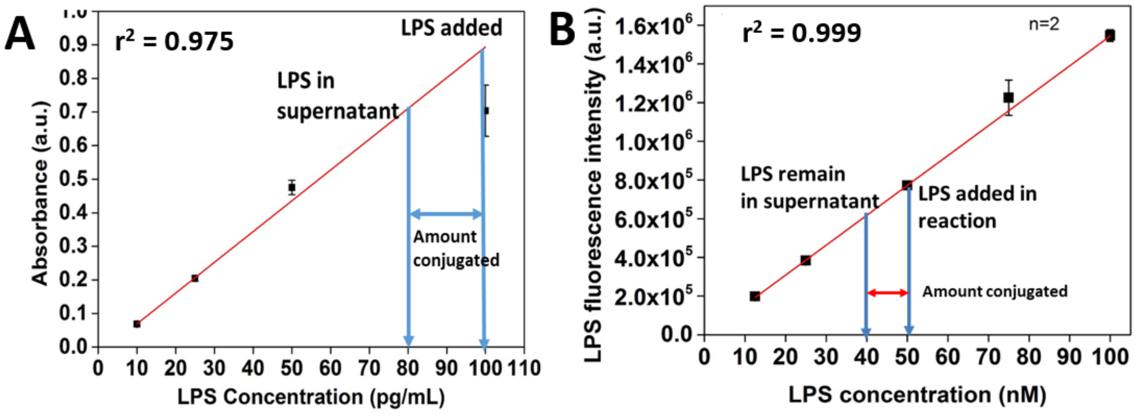

Fig. S5. Calibration curves obtained during LPS quantification in rTLR4-LPS reaction supernatants. (A) Using LAL assay and **(B)** using our fluorescence assay. All reactions were performed in 0.5% PBST, pH 7.4 (n = 3).

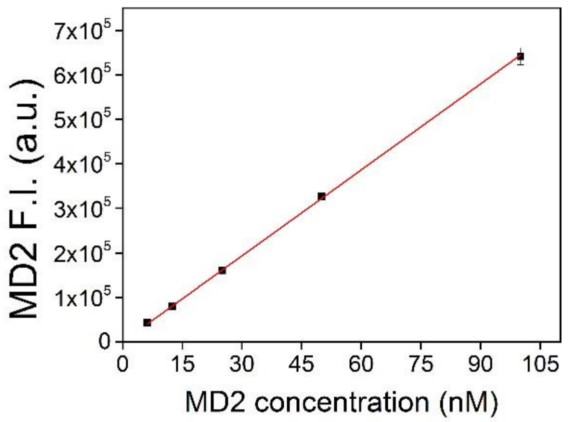

Fig. S6. Fluorescence calibration curve obtained for MD2 in 0.5% PBST, pH 7.4. (n = 3, r^2 = 0.999).

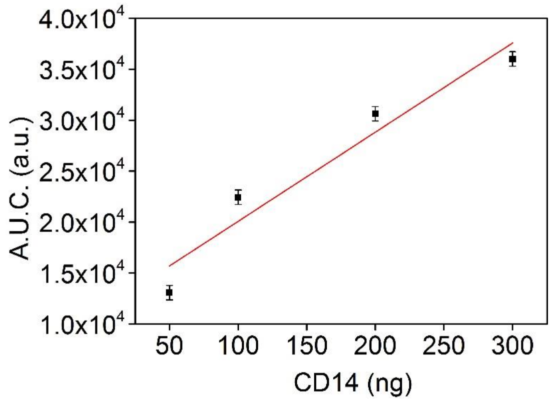

Fig. S7. Calibration curve obtained for CD14 using the silver staining procedure. (n = 3, r^2 = 0.978)
